# fNIRS-EEG BCIs for Motor Rehabilitation: A Review

**DOI:** 10.3390/bioengineering10121393

**Published:** 2023-12-06

**Authors:** Jianan Chen, Yunjia Xia, Xinkai Zhou, Ernesto Vidal Rosas, Alexander Thomas, Rui Loureiro, Robert J. Cooper, Tom Carlson, Hubin Zhao

**Affiliations:** 1 HUB of Intelligent Neuro-engineering (HUBIN), Aspire CREATe, IOMS, Division of Surgery and Interventional Science, University College London (UCL), Stanmore, London HA7 4LP, UK; jianan.chen.22@ucl.ac.uk (J.C.); yunjia.xia.18@ucl.ac.uk (Y.X.); xinkai.zhou.21@ucl.ac.uk (X.Z.); alex.thomas@ucl.ac.uk (A.T.); 2 DOT-HUB, Department of Medical Physics & Biomedical Engineering, University College London (UCL), London WC1E 6BT, UK; ernesto.vidal@ucl.ac.uk (E.V.R.); robert.cooper@ucl.ac.uk (R.J.C.); 3 Digital Health and Biomedical Engineering, School of Electronics and Computer Science, University of Southampton, Southampton SO17 1BJ, UK; 4 Aspire CREATe, Department of Orthopaedics & Musculoskeletal Science, University College London (UCL), Stanmore, London HA7 4LP, UK; r.loureiro@ucl.ac.uk (R.L.); t.carlson@ucl.ac.uk (T.C.)

**Keywords:** motor rehabilitation, brain–computer interface, functional near-infrared spectroscopy, electroencephalography, multimodal, motor imagery

## Abstract

Motor impairment has a profound impact on a significant number of individuals, leading to a substantial demand for rehabilitation services. Through brain–computer interfaces (BCIs), people with severe motor disabilities could have improved communication with others and control appropriately designed robotic prosthetics, so as to (at least partially) restore their motor abilities. BCI plays a pivotal role in promoting smoother communication and interactions between individuals with motor impairments and others. Moreover, they enable the direct control of assistive devices through brain signals. In particular, their most significant potential lies in the realm of motor rehabilitation, where BCIs can offer real-time feedback to assist users in their training and continuously monitor the brain’s state throughout the entire rehabilitation process. Hybridization of different brain-sensing modalities, especially functional near-infrared spectroscopy (fNIRS) and electroencephalography (EEG), has shown great potential in the creation of BCIs for rehabilitating the motor-impaired populations. EEG, as a well-established methodology, can be combined with fNIRS to compensate for the inherent disadvantages and achieve higher temporal and spatial resolution. This paper reviews the recent works in hybrid fNIRS-EEG BCIs for motor rehabilitation, emphasizing the methodologies that utilized motor imagery. An overview of the BCI system and its key components was introduced, followed by an introduction to various devices, strengths and weaknesses of different signal processing techniques, and applications in neuroscience and clinical contexts. The review concludes by discussing the possible challenges and opportunities for future development.

## 1. Introduction

Motor disability, also known as motor impairment, refers to a reduction or complete loss of function in one or more body parts [1]. Motor disability can lead to decreased mobility and manual dexterity, motor coordination difficulties, or even paralysis. A variety of medical conditions can cause motor disability, including (but not limited to) stroke, spinal cord injury, multiple sclerosis, brain injury, Parkinson’s disease, neuromuscular diseases, major orthopedic injury, cerebral palsy, and aging [2]. Rehabilitation training is one of the most important ways to treat motor disability and improve the quality of life of patients [3]. It is estimated that at least one-third of the world’s population requires rehabilitation at some point in their life due to illness, injury, or trauma [2].

Active motor training is the principal approach used to drive motor recovery in patients, which enhances the activity of the primary motor cortex via physical and occupational therapy [4] and pharmacological interventions [5]. Patients usually receive professional rehabilitation training at rehabilitation centers as well as extended training treatments (i.e., outside of the rehabilitation center) in daily life [6,7]. However, individuals with severe motor impairments, such as those who are paraplegic or quadriplegic, may face difficulties in performing therapeutic movements, making it challenging to observe external improvement and monitor the recovery process. Additionally, using physical assistance equipment (e.g., functional electrical stimulation (FES), robotic devices, and exoskeletons) may be limited due to cost, bulkiness, or a narrow focus on specific rehabilitation areas [8]. Furthermore, even those with moderate symptoms may not have access to rehabilitation training devices due to economic disparities or the unequal distribution of medical resources. Therefore, new strategies are necessary to supplement physical aids and accelerate motor recovery during rehabilitation therapy.

Brain–computer interface (BCI) technology offers an alternative avenue for motor rehabilitation. BCI is a direct communication pathway that measures, decodes, and translates electric, magnetic, or metabolic brain activity into commands for controlling external devices. In motor rehabilitation, patients often mentally rehearse physical movements through a process known as motor imagery (MI), which can physically alter neuronal connections within the brain to adapt to the sensory input, a process called neuroplasticity [8]. BCI can provide meaningful feedback to the central nervous during the motor rehabilitation system to help direct plasticity by measuring the change in cortical activity in response to a stimulus or when rehearsing a voluntary movement, thus helping the patient to access the motor system and facilitate rehabilitation across all stages of motor recovery [9]. Several studies have already shown that BCI is a promising way for the motor recovery of patients suffering from motor impairment [10,11,12].

Both functional near-infrared spectroscopy (fNIRS) and electroencephalography (EEG) can be used to measure relevant brain activities for BCI applications. fNIRS measures the intensity changes of near-infrared light that has travelled through the scalp and brain to monitor the brain’s hemodynamic activities [13]. EEG records the electrical signals indicating the activity of populations of neurons over a short period using multiple electrodes placed on the scalp. At present, both fNIRS and EEG technologies are commonly used in BCIs, due to their fine non-invasiveness, cost-effectiveness, portability, and potential capacity for long-term monitoring within and outside of laboratory settings [14,15,16,17]. However, fNIRS and EEG both possess inherent advantages and disadvantages. EEG offers high temporal resolution, but it has low spatial resolution and holds clear vulnerability to motion artifacts [18]. On the other hand, fNIRS can provide spatially specific signals and could be potentially more tolerant of motions compared to EEG recording [19]. Additionally, the use of diffuse optical tomography (DOT) has the ability to provide 3D imaging of cortical hemodynamic changes. The temporal resolution of fNIRS in BCIs is limited to about 1 s, due to its sensitivity to the vascular responses to increased neuronal demand [20]. Therefore, combining fNIRS with EEG is becoming a promising approach to overcome the inherent limitations of each technology and achieve complementary measurements of cerebral activity in BCIs [21].

The idea of integrating multiple signals or modalities in BCIs is called multimodality. A multimodal BCI facilitates the integration of information from multiple sources, enabling the improved measurement and decoding of users’ brain activity. Specifically, the fNIRS-EEG BCI can simultaneously measure electrical and hemodynamic activities in the brain. Several studies have shown that this integration can improve the classification accuracy of the command translated from the brain activity [21,22,23,24] and enhance the understanding of the neurovascular relationship, aiding patients with motor disabilities in neurorehabilitation [23]. However, some challenges remain to be solved to achieve a fully integrated fNIRS-EEG BCI suitable for motor rehabilitation [14], including the device wearability, signal processing method, and multimodal system integration.

In this review, our primary focus is on the advancements and potential of fNIRS BCI systems, with a special emphasis on how their integration with EEG can lead to significant improvements in wearable BCI technologies. We aim to explore the synergistic effects of this combination, particularly in the context of motor rehabilitation, highlighting how the fusion of fNIRS and EEG can enhance the efficacy and applicability of BCIs for individuals with motor impairments. The combination of fNIRS and EEG systems has made extraordinary progress, covering areas of instrumentation, brain–computer interfaces, brain function, and gait rehabilitation. Detailed reviews are available in [25,26,27,28]. While previous reviews have primarily focused on the general applications of multimodal BCIs, we provide an analysis of fNIRS-EEG BCIs specifically aimed at motor rehabilitation, including the development, limitations, necessary improvements, and current translational applications. The remainder of this paper is structured as follows: Section 2 describes the methodology of our literature review and the criteria for identifying and categorizing published works. Section 3 illustrates the principle of fNIRS-EEG BCIs and the commonly used devices. Section 4 reviews the current methods utilized in (pre)signal processing, feature extraction, and classification. Section 5 summarizes the current translational applications of fNIRS-EEG BCIs in motor rehabilitation. Finally, we summarize the key point of our review and discuss the areas of future advancement in Section 6.

## 2. Identified Publications

Google Scholar and Web of Science search engines were used for keyword searches: For fNIRS BCI: [Brain Computer Interface (BCI)] AND [Motor imagery (MI)] AND [Functional Near-Infrared Spectroscopy (fNIRS)] OR Electroencephalograph (EEG) OR fNIRS-EEG] AND [Stroke OR Motor-rehabilitation OR Motor-recovery OR Gait disorders OR rehabilitation]; For fNIRS-EEG BCI: [Brain Computer Interface (BCI)] AND [Motor imagery (MI)] AND [[Functional Near-Infrared Spectroscopy (fNIRS) AND Electroencephalograph (EEG)] OR [fNIRS-EEG]] AND [Stroke OR Motor-rehabilitation OR Motor-recovery OR Gait disorders OR rehabilitation]. Following the keyword searches, we retrieved a pool of articles from both search engines. These articles were then subjected to a meticulous manual screening process. During this assessment, we specifically selected articles that described either fNIRS-based BCIs or fNIRS-EEG-based BCI technologies and had discussions or clear potential clinical implications for motor rehabilitation. Conversely, we excluded articles that were limited to studies on healthy subjects without a rehabilitation context or those that did not provide clear evidence of the use of fNIRS in BCIs. This search strategy resulted in 23 key publications summarized in Table 1 and Table 2. More details can be found in Tables S1 and S2.

While the methods and applications described in these works vary significantly, we have attempted to group all the systems into two categories based on their modality. There are 11 fNIRS-BCI studies (including combination with eye trackers) and 12 fNIRS-EEG BCI studies. Regarding signal classification methods, there are twenty-two involving conventional machine learning (ML) algorithms and six utilizing deep learning (DL) methods. In the context of motor rehabilitation, based on the target limb, we divided these publications into upper and lower limb, which are ten and four studies, respectively, discussed in Section 5.

## 3. BCI Systems

In this section, we first provide a more detailed description of the components of a multimodal BCI system, then focus on different devices for brain signal acquisition, including fNIRS, EEG, and the combined modality. As the initial step involved in building a BCI system, the choice of adequate devices to acquire signals is of great importance.

### 3.1. Typical BCI Structures

**Figure 1:**
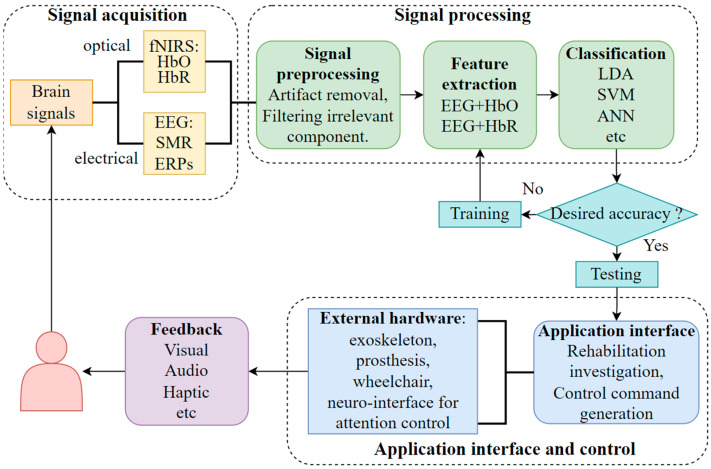
Typical hybrid—BCI system (HbO—oxyhemoglobin; HbR—deoxyhemoglobin; SMR—sensory motor rhythm; ERPs—event-related potentials; LDA—linear discriminant analysis; SVM—support vector machines; ANN—artificial neural network).

A typical multimodal BCI system consists of five stages [25]: signal acquisition, signal pre-processing, feature extraction, classification, and the application interface. Figure 1 depicts a schematic of a hybrid fNIRS-EEG BCI. The brain activity signals are first collected and amplified by the fNIRS and EEG devices, before going through signal filtering and pre-processing. After the pre-processing, distinct features of the signals are extracted. Some commonly used fNIRS and EEG features are signal peak, slope, mean, kurtosis, skewness, and power spectrum density [50]. These features are fed into a classifier in the next stage. At the final stage, the classified signals are transmitted to a computer or other devices to generate the control commands for the hardware (exoskeleton, prosthesis, wheelchair, neuro-interface for attention control, etc.). In the feedback loop, the form of presentation can be abstract (such as a moving bar on a screen), embodied (such as a real-time display of controlled activity based on virtual reality (VR)), or somatosensory representations delivered through haptic, robotic, or neuromuscular electrical stimulation (NMES) systems [1].

Speed, accuracy, ease of use, and length of training period are some of the key criteria for assessing BCI systems for MI. The MI-based BCIs can form an information channel for motor-impaired people, as it can be visualized and provide feedback to both the patient and the therapist. Furthermore, patients without mobility can gain control of external devices by utilizing MI in BCI systems. Common MI tasks include imagining actions such as squeezing a soft ball, finger tapping, foot tapping, and hand grasping.

### 3.2. BCI Hardware

#### 3.2.1. fNIRS Devices (Fiber/Fiberless)

**Figure 2:**
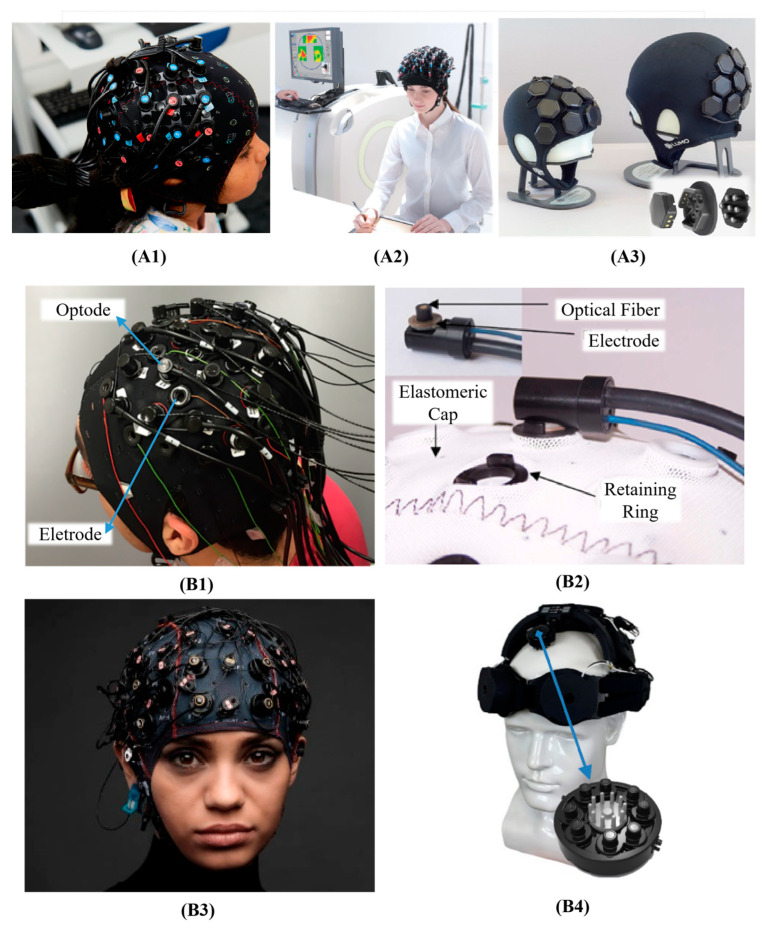
(**A**) Examples of some fNIRS devices. (**A1**) NIRScout (NIRx GmbH, Berlin, Germany). This figure is taken with permission from [57]. (**A2**) LABNIRS system (Shimadzu Corporation, Kyoto, Japan). This figure is taken with permission from [58]. (**A3**) LUMO (Gowerlabs Ltd., London, UK). This figure is taken with permission from [56]. (**B**) Examples of some fNIRS-EEG designs. (**B1**) An NIRScout cap completely mounted (with EEG electrodes, fNIRS sources, and detectors). This figure is taken with permission from [44]. (**B2**) A NIRx fNIRS/EEG cap using collocated passive EEG electrodes with a fNIRS probe. This figure is taken with permission from [59]. (**B3**) g.GAMMAcap fNIRS system: designed to mount g.SENSOR fNIRS together with g.SCARABEO electrodes or g.SAHARA hybrid electrodes (g.Tec medical engineering GmbH, Austria). This figure is taken with permission from [60]. (**B4**) Wearable Sensing’s wireless DSI-EEG + fNIRS system: a total of 8 sensor pods on the system, and each pod has 1 dry EEG electrode in the middle, 4 emitters, and 4 detectors surrounding. (Wearable Sensing, San Diego, CA, USA). This figure is taken with permission from [61].

The quantity of research related to fNIRS has experienced a significant surge over the past two decades, concomitant with the increasing accessibility of commercially available fNIRS systems [51]. The benchtop fNIRS devices, such as NIRScout (NIRx GmbH, Berlin, Germany) in Figure 2(A1) [24,44,48,52], DYNOT (NIRx Medical Technologies, New York, NY, USA) [36,39,40], NirScan (Danyang Huichuang Medical Equipment Co., Ltd., Danyang, Jiangsu, China) [47,53], and the LABNIRS system (Shimadzu Corporation, Kyoto, Japan) in Figure 2(A2) [37], are usually developed with bulky control electronics and cumbersome fiber bundles.

Wearable fNIRS devices can be achieved by fiberless design and lightweight electronics, such as Nirsmart series (Nirsmart, Danyang Huichuang Medical Equipment Co., Ltd., Danyang, Jiangsu, China) in Figure 2(A3) [54]. The wearability of the device provides the patients with more flexibility and comfort during motor rehabilitation. More recently, Gowerlabs Ltd. (London, UK) have developed a series of high-density fNIRS/diffuse optical tomography (DOT) system [55] with high sampling density across the entire scalp called LUMO [55,56]. As a fiberless, truly wearable, and user-friendly system, LUMO introduces a modular design consisting of individual hexagonal sensor tiles (Figure 2(A3) Right), that can be effortlessly connected into a cap at various locations. The versatility and scalability of LUMO’s design can make it a valuable tool for high-quality 3D neuroimaging and high-density BCIs. 

#### 3.2.2. fNIRS-EEG Devices

**Figure 3:**
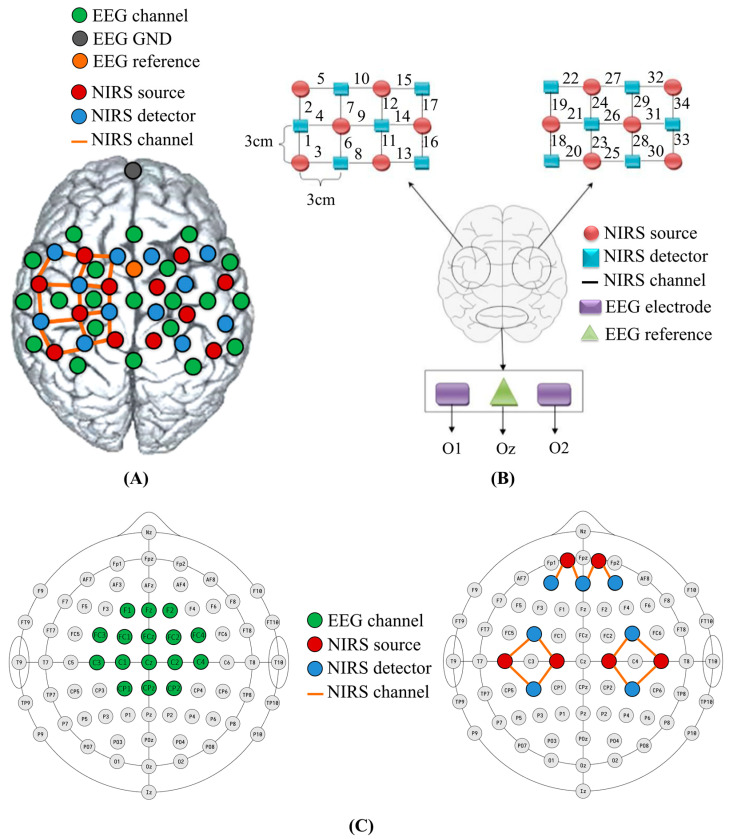
Examples of some fNIRS-EEG configurations in BCI studies. (**A**) A cohesive configuration of optodes and electrodes. This figure is taken with permission from [44]. (**B**) Electrode and optode placement of EEG (for visual frequency change) and NIRS (for left and right motor imageries). Numbers indicates fNIRS channels. This figure is taken with permission from [40]. (**C**) Illustration of a BCI–robot system. The subfigure on the left illustrates 16 EEG electrodes used in experiments. The subfigure on the right illustrates a fNIRS configuration with 12 channels. The probes are located over the prefrontal cortex (bilateral cortex near FPZ), the primary motor cortex (M1) (bilateral). This figure is taken with permission from [53].

An ideal BCI system should be able to effectively gather real-time information about brain activity, with a quick setup time and long-term stability [27]. As the diversity of commercial devices increases, numerous fNIRS-EEG studies have used a cap that covers the entire head with a cohesive configuration that includes both optodes and electrodes [44] (Figure 3A) or applied different modalities to separate positions on the scalp [40] (Figure 3B). 

Some researchers have designed their own cap to better accommodate a target brain area or a specific task. For example, Koo et al. designed a unique sensor frame with each part holding three electrodes, one detector, and four sources on one hemisphere of the scalp [42]. They combined Imagent (ISSInc., Champaign, IL, USA) [42,46] and g.MOBIlab+ (g.Tec medical engineering, Schiedelberg, Austria) [40,42] to concurrently record fNIRS and EEG. Another common solution for fNIRS-EEG BCIs is to combine commercially available individual systems on a single headcap to collect fNIRS and EEG signals. For example, NIRScout can be integrated with other EEG devices such as Brain Products [24,48,52] and microEEG (BioSignalGroup, Brooklyn, NY, USA) [44]. Specifically, the sensor placement for EEG can be performed in two distinct ways: adjacent positioning (Figure 2(B1)) or co-located measures (Figure 2(B2)). While adjacent positioning allows for experiments with any type of EEG electrode and reduces setup time, co-located measures are restricted to ring electrodes and necessitate transparent gel.

To our knowledge, there is no fully integrated fNIRS-EEG device available to be applied in motor rehabilitation [14]. However, there have been a few attempts made in this direction. Some companies have adopted an add-on modular approach to facilitate the construction of multimodal systems. For example, g.Tec has developed the g.SENSOR fNIRS device (g.Tec medical engineering GmbH, Schiedlberg, Austria), which consists of eight channels and can be used in conjunction with g.Tec’s EEG amplifiers, such as the g.USBamp, allowing for simultaneous recording of both EEG and fNIRS signals. It can be mounted with a magnet holder onto the g.GAMMAcap fNIRS system (Figure 2(B3)), which includes one set of optode holders located on the frontal cortex and another set situated over the sensorimotor cortex. The device is equipped with low-power optodes for the frontal cortex and high-power optodes for the sensorimotor cortex. The wireless DSI-EEG + fNIRS system (Figure 2(B4)) developed by Wearable Sensing (San Diego, CA, USA) represents a significant advancement, as it is the first truly hybrid system capable of simultaneously recording dry EEG and fNIRS signals from the same location. Of note, besides the two solutions mentioned above, some BCIs utilize EEG signals for decoding brain activities while using the fNIRS device to monitor cortex activations (Figure 3C) [53,54].

## 4. Signal Processing in fNIRS and fNIRS-EEG-Based BCIs

Following an overview of the hardware, this section focuses on the signal processing methods and study outcomes in both fNIRS-based BCIs (Section 4.1) and fNIRS-EEG-based BCIs (Section 4.2) to evaluate the improvement in performance that results from the hybridization of these two modalities. In each subsection, we cover the topics of signal acquisition and pre-processing, channel selection and feature extraction, and classification, which are all essential elements in a BCI system. An overview of signal processing methods for fNIRS-BCIs and fNIRS-EEG BCIs on motor-related tasks are presented in Table 1 and Table 2, respectively, with chronological order.

### 4.1. fNIRS-Based BCIs

#### 4.1.1. Data Pre-Processing

The acquired fNIRS signals usually contain various noises, including instrumental noise, experimental noise, and physiological noise [20,25]. These noises can obscure functional brain activity and should be eliminated before converting the raw optical densities into concentration changes of oxyhemoglobin (HbO) and deoxyhemoglobin (HbR).

A variety of methods can be implemented to eliminate noise in fNIRS-BCI systems. These include band-pass, adaptive, Kalman, Wiener, Gaussian, and hemodynamic response filters (hrf), along with finite impulse response techniques [36]. Additionally, advanced analytical methods like principal component analysis (PCA) and independent component analysis (ICA) are employed [25]. Temporal filters are also in use, such as the moving average convergence/divergence (MACD) filter for real-time band-pass filtering. This method, which calculates the difference between two exponential moving averages (EMA), has been successfully tested in several studies [37]. The MACD filter was notably utilized in an fNIRS-BCI system for stroke recovery, aiming to remove global trends and physiological noise from respiration and cardiac movements. 

Furthermore, wavelet transform (WT)-based methods offer an alternative for noise reduction [20,35,38]. Continuous wavelet transforms (CWT), applied with a soft thresholding rule [62], capture significant fNIRS signal features by localizing the time-frequency characteristics of non-stationary signals. Discrete wavelet transforms (DWT) analyze wavelet coefficients (WCs) and their temporal variations for each channel. One application involved developing a detrending algorithm using the wavelet minimum description length (MDL) algorithm in an fNIRS-BCI study for lower limb movement preparation in chronic stroke patients [33]. This pre-processing stage decomposes the signal into components of global trends, hemodynamic signals, and noise, thereby mitigating the occurrence of false or extraneous components.

Building upon these noise reduction strategies, it is presumed that recorded fNIRS data comprise both task-evoked and task-independent components, which can be effectively separated [63]. In this context, data-driven methods such as ICA, PCA, or task-related component analysis play a crucial role in isolating real functional activity. Notably, PCA has been specifically utilized to correct sections identified as motion artifacts [64]. Moreover, motion detection in these studies can be conducted manually or through the integration of motion sensors or tracking systems. An illustrative example of this is seen in a study focusing on movement intention for gait rehabilitation. In this research, electromyography (EMG) was employed to differentiate between voluntary and involuntary activities, thereby aiding in pinpointing the initiation of movement [33].

#### 4.1.2. Channel Selection and Features Extraction

##### Channel Selection

Before extracting features, selecting the appropriate channels is essential to simplify the analysis and ensure reliable data. A study involving six neurologically intact participants found a positive correlation between the number of channels used (up to nine) and classification accuracy [34]. However, it was observed that the optimal classification outcomes were achieved with a smaller quantity of channels, also emphasizing the significance of the quality for the selected channels. Channels with a high coefficient of variation (standard deviation divided by the mean) are typically considered unsuitable and excluded from the analysis [64].

However, the effectiveness of channel selection may be contingent upon the scale of measurement, specifically, the number of channels produced. In a follow-up study to [34], ref. [37] explored a wireless brain–computer interface (BCI) with a robotic hand orthosis aiding motor-impaired patients. They used fNIRS to observe the motor cortex and an LDA classifier to predict hand postures based on mean and slope features. The study evaluated how feature window length and feature vector types (‘preserving’ vs. ‘averaging’ channels, and ‘all’ vs. ‘criterion-selected’ channels) influenced the fNIRS-BCI’s accuracy and response time. Channels were selected based on the contrast-to-noise ratio (CNR), favoring channels with positive CNR for HbO and negative for HbR. The optimal results were with HbO + HbR signals and ‘preserving channels’, achieving 71.54% accuracy and 2.9 s latency. However, using criterion-selected channels did not outperform using all channels. The findings suggest the need for more selection criteria and indicate that channel reduction might reduce computational resources without compromising performance.

##### Features Extraction

Although feature selection also depends on different tasks, the mean and slope values of HbO, HbR, and total hemoglobin (HbT) concentrations are commonly used as features in fNIRS-based BCI studies [33,37]. In the early detection of a task, the slope feature seems significant [44]. It has been shown that HbO performs more robustly than HbR and HbT for assessing task-related cortical activation [25], and may yield higher accuracy for MI tasks [24]. To account for the temporal changes in the hemodynamic response, a fifth-order polynomial regression (PR) algorithm was utilized to analyze the changes in the concentration of HbO responding to hand-tapping tasks, with the coefficients of the regression curve serving as features [32]. This approach offers the advantage of numerical analysis and visualization through graphical representations.

#### 4.1.3. Classification

In some cases [34,43], researchers have also utilized the HbD signal (the difference between HbO and HbR, also known as cerebral oxygen exchange (COD)). For instance, in an fNIRS-based robotic hand rehabilitation system, the HbD signal was used together with HbO [34]. Since HbO signals have a broader dynamic range than HbD, and this range can vary between channels, data standardization and scaling are necessary. Standardization was performed by subtracting the mean value from each signal and dividing it by the standard deviation to remove outliers, such as large peaks in the HbO signal. Each standardized signal was then scaled to a range between 0 and 1. Some other common time domain features besides signal mean and slope are signal skewness, kurtosis, signal variance, and signal peak [12,36,65,66]. They are usually combined with mean or slope, and their performance varies for different tasks and applications. Several studies in fNIRS also proposed using filter coefficients as classification features, such as Kalman filtering, WT, and recursive least-square estimation [12,25,65,66]. The aim of classification is to accurately classify the brain state based on the extracted features, and it is a fundamental step in the processing of BCIs. Some frequently used methods include linear discriminant analysis (LDA), support vector machine (SVM), hidden Markov model (HMM), k-nearest neighbor (k-NN), Naïve Bayes, and quadratic discriminant analysis (QDA) [31,36].

Khan et al. presented an fNIRS-BCI framework for the control of prosthetic legs, which compared the performance of different combinations of filters, features extracted, and classifiers [36]. Six different filters (Kalman, Wiener, Gaussian, hrf, band-pass, finite impulse response) were evaluated and six different features extracted from HbO were used for classification (signal mean, signal slope, signal variance, slope kurtosis, signal peak, and signal skewness). Five different classifiers (k-NN, QDA, LDA, naïve Bayes, SVM) were also tested. The results showed that the combination of SVM and hrf-processed signals achieved the highest classification accuracy. However, the combination of LDA and six features was adopted for online BCI due to its low computational cost and execution delay. Another study also demonstrated that LDA outperformed SVM in real-time data processing [34]. Although SVM had a slightly higher average classification accuracy because of its generalization capability to new data, its high computational cost was a concern. In this particular case, as the system was designed for the online application of robotic hand rehabilitation, LDA was selected for its mathematical simplicity and low computational load, allowing for faster implementation. However, SVM remains one of the most resource-efficient ML methods for classification purposes [27].

The development of artificial neural networks (ANN) also brings new inspiration to the classification of brain activities. In 2013, a study compared the performance of SVM and a three-layer ANN in classifying left-hand and right-hand-tapping tasks [32]. The small sample size of only three subjects makes it difficult to determine which method outperforms the other, although SVM was found to be faster in terms of recognition time. These findings suggest that shallow neural networks may not have a clear advantage over ML algorithms like SVM. Therefore, researchers are also exploring the feasibility of using deep neural networks (DNN) in fNIRS-BCIs.

##### Deep Learning

**Figure 4:**
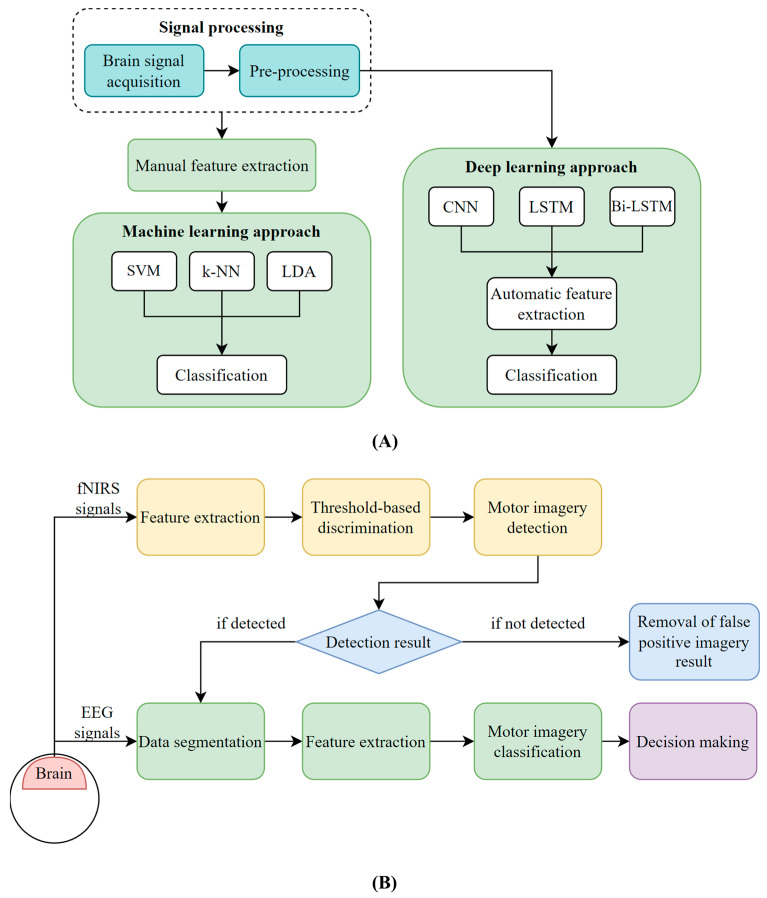
Examples of some signal processing pipelines in BCI systems. (**A**) Comparison of time-series fNIRS signal processing of conventional ML and DL algorithms. This figure is taken and modified with permission from [39], with an application for classifying walking and rest tasks in gait rehabilitation. (**B**) The procedure of sequential data processing in hybrid BCIs. Reprinted/adapted with permission from Ref. [42]. 2023, Elsevier.

Based on the previous discussions, it is evident that the process of classifying fNIRS signals using conventional ML methods involves a number of steps, such as noise removal, channel selection, local and global feature extraction, dimensionality reduction, and feature space combination. However, these steps can result in biases and overfitting of the data, and a considerable amount of time is required for data mining and pre-processing to address these limitations [39]. As illustrated in Figure 4A, DL methodologies can extract features automatically and perform classification seamlessly in a single step.

In 2017, a study utilized convolutional neural networks (CNN) to classify fNIRS signals during right-hand and left-hand motor execution (ME), as well as the rest state, achieving a higher accuracy of 92.68% compared to SVM and ANN which achieved 86.19% and 89.35%, respectively [35]. The convolutional filters in the CNN model also produced better-discriminated features compared to conventional features and hemodynamic response signals. However, the CNN model required a longer computational time during training and testing compared to the ANN and SVM models, indicating the need for further improvements. 

A 2020 study compared the performance of a CNN with conventional ML algorithms, such as SVM, multilayer perceptron (MLP) neural network, and projection-based learning in a meta-cognitive radial basis function network (PBL-McRBFN), for classifying different motor tasks, including right-fist clenching, left-fist clenching, right- and left-foot tapping, and rest using fNIRS signals [38]. The CNN outperformed the other algorithms with an average classification accuracy of 72.35 ± 4.4%. This study demonstrates the potential of DL approaches, specifically using the spectrogram representation of fNIRS signals, for developing BCI applications. CNN-based time-series classification (TSC) methods were evaluated and compared to conventional ML methods, such as SVM, for their ability to classify fNIRS-BCI signals [67]. The CNN-based methods outperformed the ML methods in terms of classification accuracy, particularly for left-handed and right-handed MI tasks, achieving up to 98.6% accuracy. These findings indicate that utilizing CNN-based TSC methods can notably enhance BCI performance while also establishing the basis for downsizing and increasing the portability of training rehabilitation devices.

A recent study presented a BCI framework for gait rehabilitation using both DL and ML techniques [39]. The DL algorithms used in the study were CNNs, long short-term memory (LSTM), and bidirectional LSTM (Bi-LSTM), which achieved an average classification accuracy of 88.50%, 84.24%, and 85.13%, respectively. These results outperformed conventional ML algorithms (SVM, k-NN, LDA) by at least 10%. The classifiers’ control commands can initiate and terminate the gait cycle of the lower limb exoskeleton, providing effective assistance in gait training for the elderly and disabled.

### 4.2. fNIRS-EEG-Based BCIs

In the context of online BCI systems utilized for rehabilitation purposes, it is important to prioritize not only high accuracy but also the ability to quickly translate correct brain activity, so as to provide timely feedback. The integration of fNIRS and EEG in monitoring brain activity in BCIs has yielded promising improvements, including better performance in classification accuracy [24], an increased number of control commands [41,65], and a faster response time [45]. In this subsection, we present different approaches for data pre-processing and classification when integrating multimodality and demonstrate the capability of these improvements to contribute to motor rehabilitation BCI systems.

#### 4.2.1. Pre-Processing and Improved Reliability of Hybrid Signals

EEG features are extracted from frequency bands that are associated with specific brain activities, including delta (<4 Hz), theta (4–7 Hz), alpha (7–12 Hz), mu (8–13 Hz), beta (12–30 Hz), and gamma (>30 Hz) [68], with the mu and beta bands commonly referred to as sensorimotor rhythms (SMR). During the execution or imagination of a motor task, the mu–beta activity is suppressed in related brain regions, resulting in a proportional decrease in power; this process is event-related desynchronization (ERD). In the context of motor rehabilitation BCIs, the alpha–beta band is of primary interest, and various filtering techniques can be applied to eliminate frequencies that are not within our interest.

Alongside frequency and temporal filters, which eliminate undesirable frequency components and reduce noise through signal averaging or weighting over time, spatial filters are also utilized, particularly in conjunction with EEG-fNIRS fusion approaches, to obtain more comprehensive spatial information. In previous studies [24,42,44], the common spatial patterns (CSP) method was used to apply spatial filters to separate neural signals originating from distinct brain regions. In [42], the spatial filters were tailored for decoding left and right MI events separately. Two-dimensional signals were extracted from each EEG segment by applying spatial filters. Subsequently, log-scaled variance and power spectral densities of different frequency bands were computed from each signal. In [44], CSP for EEG was regularized with information from all participants and optimized using genetic algorithms to classify right–left and arm–hand. The CPS approach also has the potential to attenuate signals originating from brain areas that are influenced by noise or artifacts.

In hybrid BCI systems, channel selection is equally critical as in fNIRS standalone systems. One study in 2017 employed the general linear model (GLM, a popular method that fits the expected hemodynamic response to the measured fNIRS signal) [45] to use the spatial information from fNIRS to identify the single fNIRS channel and EEG channel on each hemisphere which yielded the most significant contrast between the two classes of motor tasks. The benefit of this procedure is the enhancement of classification accuracy with the minimization of the number of channels, thereby enhancing the efficiency of data processing and accelerating the response time of the BCI.

##### Features and Selection Methods

The power spectral density method, which assesses the signal strength as a function of frequency, is widely utilized in fNIRS-EEG studies for feature classification [50]. Some other studies employed EEG processing features, including logarithmic band power [12] and wavelet approximation coefficients [45]. The logarithmic band power is estimated by taking the logarithms of the power of various frequency bands of EEG data. The discrete wavelet transform (DWT) technique in EEG, similar to the wavelet transform used in fNIRS, can decompose the time-series data of each channel into multiple layers. A study by [45] suggests that the wavelet approximation coefficients derived from the final layer of the DWT process capture the predominant power of event-related oscillations in brain activity, which is left- and right-hand movements in this particular case.

For hybrid BCIs, a combination of the signal peak and mean fNIRS signals and the highest band powers of EEG signals could be desirable [12]. Other studies also used the time-frequency phase feature (including power, instantaneous amplitude (IA), instantaneous phase (IP), and instantaneous frequency (IF)) extracted from EEG to combine with HbD for hybridization [43]. It is worth noting that optimal classification may require different features depending on the specific tasks being performed, as demonstrated in [24]. In comparison to EEG signals alone, the average classification accuracy of MI tasks using EEG and HbO features increased from 78.2% to 83.2%. In contrast, higher accuracy was achieved for ME tasks using EEG and HbR features, improving from 90.8% to 93.2%.

A growing hemodynamic feature of interest is the initial dip, a metabolically linked phenomenon noticed early in 1990, where the HbR concentration starts to increase in 2 s upon the activity, followed by a later and more pronounced decrease [45]. This method can overcome the inherent delay for the hemodynamic response hindering the efficiency of a real-time fNIRS-based BCI application because of its rapid evolution in the face of stimuli. In 2016, Zafar et al. used vector-based phase analysis to show that detecting and classifying the initial dips is feasible in fNIRS despite the relatively low amplitude [69]. R. Li et al. also used initial dip information with PCA performed before feature extraction to further remove artifacts in the classification of left- and right-hand movements [45]. This study holds great significance in extending the motor recovery applications of BCI systems since it is the first to utilize fNIRS spatial information for channel selection and integrate early temporal information from both fNIRS and EEG to improve the system’s transfer rate while maintaining decent performance. By providing instant feedback, this approach offers the potential to enhance the user’s experience and recovery outcomes.

Last but not least, heuristic methods, including genetic algorithms, are widely used in addressing optimization problems, which can also be employed for signal processing in BCI. The implementation of genetic algorithms comprises selecting features using LDA as a fitness function [70], or it can be combined with SVM to determine the optimal feature combinations [71]. 

##### Sequential Data Processing

An alternative approach to combining fNIRS and EEG, besides a joint feature space, is to delay the decision-making process by incorporating fNIRS into the framework of an EEG-based BCI. In practical BCI applications, false positive classifications can pose a potentially dangerous risk, making it crucial to eliminate such errors.

Koo et al. employed a combination of the NIRS and EEG systems for a self-paced BCI system on online MI [42]. As shown in Figure 4B, a threshold in the NIRS system regarding the temporal MI region was used to detect the occurrence of a motor intention. Then, the EEG signals in that region were employed to differentiate the type of MI with a linear-SVM classifier. In the context of shared control for BCIs, this approach is also referred to as contextual gating [72]. Within the framework of gating, an initial command can be either accepted or rejected based on internal or external contextual factors. The parameters for the threshold and classifier were estimated from the preceding training sessions and later applied in the online session. This approach may be more advantageous when there is a need to perform challenging motor rehabilitation movements or a heightened emphasis on training safety. A study by Buccino et al. also implemented a similar approach, where the initial classification stage distinguishes between the resting and movement states of the user in an asynchronous paradigm [44]. Subsequently, the second stage of classification is triggered only upon detection of movement and classifies the specific task as either right–left or arm–hand. The aforementioned paradigm has the potential to enable real-time communication between the user and the system and expand the number of classes beyond two.

#### 4.2.2. Classification for Hybrid fNIRS-EEG

##### Conventional Machine Learning Classification Algorithms 

In MI tasks, the EEG signal exhibits less vulnerability to contamination from EMG signals when compared to ME tasks. This particular characteristic of EEG offers an advantage in capturing rapid and dynamic information, thereby contributing to enhanced temporal precision in classification. Conversely, changes in hemoglobin levels, as detected by fNIRS or DOT, typically exhibit a slower onset and longer duration compared to the electrical signals captured by EEG. Consequently, fNIRS or DOT can provide supplementary benefits in terms of spatial localization and improved overall robustness of the classification. However, the issue of signal delay in fNIRS should be carefully addressed in real-time BCI systems, and further improvements are necessary before enabling online applications for motor rehabilitation.

Classical ML techniques, such as LDA and SVM, are characterized by simplicity in training and a higher potential to achieve a robust model [27]. LDA is the most commonly used classification method in fNIRS and hybrid fNIRS-EEG studies [12], which is highly suitable for online BCI systems. For example, in [24], LDA was used as a meta-classifier, whose weights are re-estimated within each cross-validation step to avoid bias in the generalization error estimation. However, linear decision boundaries may not effectively separate non-linearly separable classes. Additionally, in cases where the number of observations exceeds the number of features, LDA might perform differently than desired. SVM is another widely adopted pattern recognition technique for brain signal classification. It has been used in various fNIRS-EEG studies [40,42] and has demonstrated comparable or even superior performance compared to the LDA in some instances [46].

##### Deep Learning Classification Algorithms

Nevertheless, the aforementioned ML methods will increase linearly in computational cost as the size of the data set expands, making them suitable for relatively small data sets with moderate standard deviations [27]. The use of DNNs in the classification of cognitive events based on fNIRS and EEG signals can result in significant improvements compared to ML methods such as LDA and SVM. This is because DNNs can perform complex, non-linear transformations and classifications, leading to unprecedented outcomes when applied to signals [46]. The classification of cognitive events presents a high-dimensional pattern classification problem with a relatively limited number of training patterns. Conventional classification methods require prior feature selection before training a model, while DNNs and CNNs can be trained directly, bypassing the need for feature selection and making them well-suited for learning from raw data [73]. 

In 2018, a DL algorithm for BCI classifications in a combined fNIRS-EEG framework was first implemented for a guided left- and right-hand MI task [46]. Two factors were considered: recording modality (EEG, fNIRS, EEG + fNIRS) and classification algorithm (LDA, SVM, DNNs). The utilization of fNIRS in conjunction with EEG demonstrates a notable improvement in average accuracy compared to the individual modalities, with an increase in accuracy from approximately 70% to 83.28%. For hybrid classifiers, DNN showed a significant increase in accuracy compared to SVM and LDA, of 5.28% ± 1.42% (paired *t*-test, t = 3.71, df = 14, *p* < 0.05, Bonferroni corrected) and 11.79% ± 2.00% (paired *t*-test, t = 5.89, df = 14, *p* < 0.05, Bonferroni corrected), respectively. The potential influence of different head coverage of electrodes and optodes was also considered in the experiment. The results obtained using only 16 electrodes around the motor cortex were comparable to those obtained using the entire EEG headset, with less than 1% variability. The same conclusion was drawn from the results of fNIRS, which demonstrated the ability of DNNs to select relevant electrodes/optodes of interest. Chiarelli et al. also attempted to implement CNN in their study, but the performance was not as strong as the fully connected feed-forward DNN. Possible explanations for this outcome were provided, including a low number of optodes and limited training data.

In 2020, CNNs were utilized for classifying workload memory tasks [73]. In the work of M. Saadati et al., the most favorable outcome was obtained with a three-second window and the exponential linear unit (ELU) activation function. For this configuration, CNNs yielded an 89% correct classification accuracy, outperforming SVMs by 7%. That same year, Ghonchi et al. employed a combination of CNNs (to extract spatial features) and recurrent neural networks (to extract temporal features) to classify both MI and MA tasks [48]. The proposed recurrent convolutional neural network (RCNN) model achieved an accuracy of over 99% by transforming sequences of chain-like signals into hierarchical three-rank tensors. These findings demonstrate the feasibility of utilizing CNN and RCNN methodologies to investigate mental workload and motor training, thereby highlighting their potential for application in rehabilitation settings.

In another recent study by Chen et al., a multi-channel fusion (MCF) approach was proposed as a means to simplify the network design in DL-based techniques for combined fNIRS-EEG classification [49]. The multi-channel fusion hybrid network (MCFHNet), which was developed based on this MCF approach, integrates depth-wise convolutional layers, a channel attention mechanism, and bidirectional long short-term memory (Bi-LSTM) layers, demonstrating exceptional performance with a mean accuracy of 99.641% in a 5-fold cross-validation of an intra-subject experiment. This study presented a novel approach for multimodal MI decoding and its potential applications in the field of upper limb rehabilitation through the implementation of MCFHNet, which exhibited strong capabilities in extracting spatiotemporal features. However, this study was based on an open fNIRS-EEG dataset [74], and further investigation is needed to assess the performance of DL algorithms in real-time BCI systems.

All these recent works demonstrate the future potential of DNNs owing to their capability of simultaneously learning features and performing classification from raw data. Although several feasibility studies have demonstrated improved accuracy with DL in the classification of fNIRS and EEG signals, there have been limited investigations into its application in the context of online motor rehabilitation BCIs in a combined fNIRS-EEG framework, making it a worthwhile area for future research.

## 5. Neuroscience and Clinical Applications

High-quality signal acquisition and advanced data processing techniques enable BCI systems to achieve satisfactory performance in imaging, assisting, augmenting, and rehabilitating human cognitive and sensorimotor functions. Specifically, BCIs can be used to help restore motor function in the following ways [4,50]: (i) real-time feedback, such as the representation of a performed action in VR; (ii) control of training devices causing actual movement with haptic, FES, or robotics feedback to develop a hybrid system during rehabilitation to gain correct movement posture [75]; (iii) control of external devices, such as wheelchair or prosthesis [76,77].

The application of fNIRS for motor recovery involves the recovery of upper and lower limb functions, balance control, and motor learning [78]. This section primarily focuses on the utilization of fNIRS-BCI and fNIRS-EEG BCI methodologies in the rehabilitation of upper and lower limb functions. These BCI approaches are commonly integrated with assistive devices to provide feedback that stimulates the pertinent muscles and brain regions, thereby facilitating the adaptation of locomotion to the training objectives.

### 5.1. Upper Limb Applications

A substantial amount of research has been devoted to the investigation of upper limb function, with a particular emphasis on hand and arm movements. It is noteworthy that the motor cortex regions responsible for controlling the left and right hands are located in separate hemispheres, thereby enabling the deciphering of commands through BCI methodologies. In 2013, a study was published which served as an initial investigation for using an EEG-fNIRS SMR-based brain switch for patients with tetraplegia and examined the difference in performance between MI and motor attempt with a task of tapping the fingers and thumb continuously [79]. Combined fNIRS-EEG modalities were proven to have significant potential especially for users who had difficulty controlling current EEG-based brain switches. The average classification performance in the patient group for ME was higher than for MI, with the highest classification of 87% accuracy found by using EEG + HbR.

Different but relevant to BCIs, a multimodal neuroimaging approach exhibits preliminary potential for monitoring and predicting post-stroke motor recovery. A novel fNIRS-informed EEG source imaging technique was devised to assess cortical activity and functional connectivity during a hand-clenching task [52]. Graph theory analysis was subsequently conducted to identify biomarkers relevant to motor function recovery and to document cortical reorganization that occurs over the course of a four-week intervention post-stroke. The aforementioned studies have demonstrated that the combination of fNIRS and EEG for the investigation of upper limb recovery has the potential to not only enhance the accuracy of task classification, but also improve the quality of neuroimaging results.

Extra rehabilitation/assistive devices can be utilized in conjunction with the BCI to facilitate motor rehabilitation. In 2020, M.A Khan et al. provided a review on MI-based BCI systems for upper limb post-stroke neurorehabilitation, which comprehensively covered different MI-BCI based strategies including FES, robotics assistance, and hybrid VR-based models [4]. The authors compared these methods in terms of their characteristics, configuration, potency, and prospects for the rehabilitation of stroke patients with upper limb impairments [4]. However, most FES-BCI systems in this review employed EEG only. In 2012, a study demonstrated that fNIRS signals generated by MI can be distinguished offline from signals induced by FES with an accuracy exceeding 70% [30]. This was the first demonstration that it is technically feasible to implement a contingent haptic-feedback fNIRS-BCI with FES.

**Figure 5:**
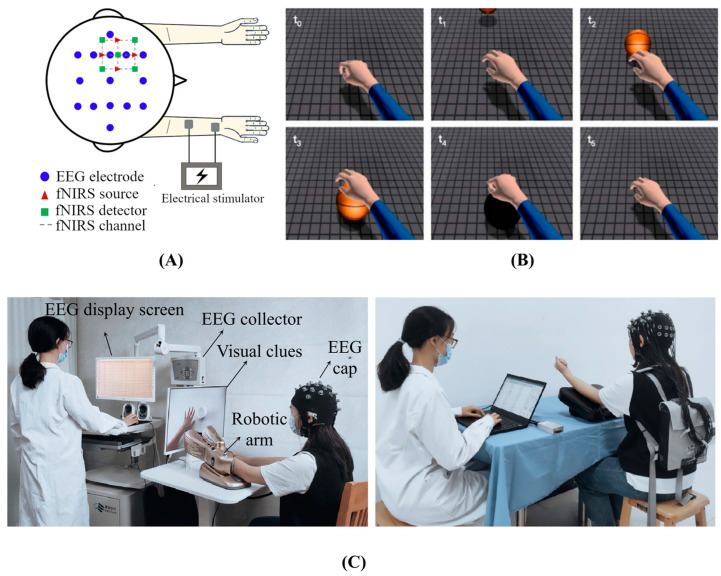
Examples of combining fNIRS and EEG measurements in BCI systems for upper limb rehabilitation. (**A**) Configuration of fNIRS-EEG with FES. The standard FES electrodes were about 10 cm apart placed at the right wrist and middle position of the forearm. Reprinted/adapted with permission from Ref. [47]. 2023, IEEE. (**B**) Ball-catching task as shown in the VR video: subjects passively watched a VR video which displayed a right hand repeatedly grasping an incoming ball (13 actions, approx. 0.86 Hz, 20 s). This figure is taken and modified with permission from [29]. (**C**) Photographs of a motor imagery brain–computer interface (MI-BCI) upper limb rehabilitation training system. The subfigures illustrate the process of using the EEG-BCI system for rehabilitation training (left) and using fNIRS to evaluate the training performance of the EEG-BCI system (right). This figure is taken with permission from [54].

FES utilizes electrical currents to stimulate nerves that innervate extremities affected by paralysis. A typical process involves the subject attempting to perform MI, followed by signal acquisition. Upon detection of the required MI events, a trigger command is then sent to a microcontroller-based hardware unit (e.g., Arduino-based) to activate the FES device. A study in 2019 investigated and compared the cortical activations during different motor training conditions (MI-BCI-FES, MI-FES, MI, and FES) measured by fNIRS and EEG [47], as shown in Figure 5A. There were significant increases in ERD patterns and cerebral hemodynamic responses for MI-BCI-FES relative to the other conditions, which also yields a relatively higher online classification performance difference.

The potential of VR-based therapy in facilitating motor learning, as well as its ability to transfer and generalize to tasks in the physical environment, has been discussed [80]. Moreover, several clinical studies have been conducted on patients to evaluate its effectiveness in rehabilitation [81]. Moreover, attempts to use wireless fNIRS with VR have also been made to study if the action-observation system was activated [29]; a series of tasks that involved imagery, observation, and imitation of hand actions were conducted [29]. Both imagery and observation tasks have been found to elicit consistently lower oxygenation changes than imitations. As in Figure 5B, participants were separated into two groups by contralateral/bilateral recording and were instructed to imagine the movement depicted in VR as their own movement. The virtual reality presented from a first-person view resulted in stronger activations in the sensorimotor network, which highlighted the potential of the VR-fNIRS instrument in neurofeedback applications. This work demonstrated the potential to control movements in the VR environment based on the brain activity recorded by the BCI system, thereby providing real-time neurofeedback.

There are commercial rehabilitation systems already on the market (recoveriX, g.tec medical engineering GmbH, Schiedlberg, Austria) incorporating motor imagery and feedback mechanisms such as utilizing functional electrical stimulation and virtual reality avatars driven by EEG signals, catering to both upper and lower limb rehabilitation needs. A study conducted with thirty-six stroke patients experiencing hemiparesis in the upper extremities utilized this system and demonstrated that the implementation of quantitative EEG tools can provide valuable insights into stroke pathophysiology and the dynamic alterations occurring within the brain during the course of rehabilitation therapy [82]. Despite these advancements, the developments of clinical rehabilitation programs for MI-based BCI systems have been partially hindered by the lack of scientifically established and standardized guidelines. The determination of the impact of frequency, intensity, and duration on neuroplasticity and clinical function is crucial for the advancement of the MI-based rehabilitation system [54]. A pilot study in 2022 [54] provided an essential reference for the formulation of clinical programs for MI-BCI training in the improvement of upper limb dysfunction. EEG data were collected during training, providing feedback with a robotic arm for motion performing (Figure 5C Left), while the fNIRS signal was recorded during the hand-grasping task for performance evaluation (Figure 5C Right). As expected, the results indicated greater cortical activation and improved BCI performance in the high-frequency group. Additionally, the within-group results revealed that after five sessions of BCI training, greater cortical activation was observed and better BCI performance was achieved in the high-frequency group, while no such effects were observed in the low-frequency group. These findings emphasize the significance of both long-term training and adequate frequency in MI-BCI rehabilitation.

An ongoing study [53] also utilized fNIRS to measure neuroplastic changes following EEG-BCI-based reward-driven hand robot practice in a single-blind, parallel-group trial involving subacute or chronic post-stroke patients with severe hemiparesis beyond 90 days after onset. However, these two studies did not integrate fNIRS and EEG signals, but rather separately used them for different purposes, i.e., using fNIRS to monitor brain activity and EEG to command the BCI.

### 5.2. Lower Limb Applications

Research about motor rehabilitation was previously limited to the upper limb [8]; however, in recent decades, more studies targeting the lower limb and gait rehabilitation have emerged. Recent technological developments indicate that fNIRS-BCI can be exploited for the rehabilitation of lower limb movement due to its great usability and reduced sensitivity to head motion artifacts [33]. Furthermore, several studies suggest that there may be a shared mechanism that influences upper and lower limb recovery simultaneously, regardless of which limb is selected for rehabilitation therapy [8,50].

**Figure 6:**
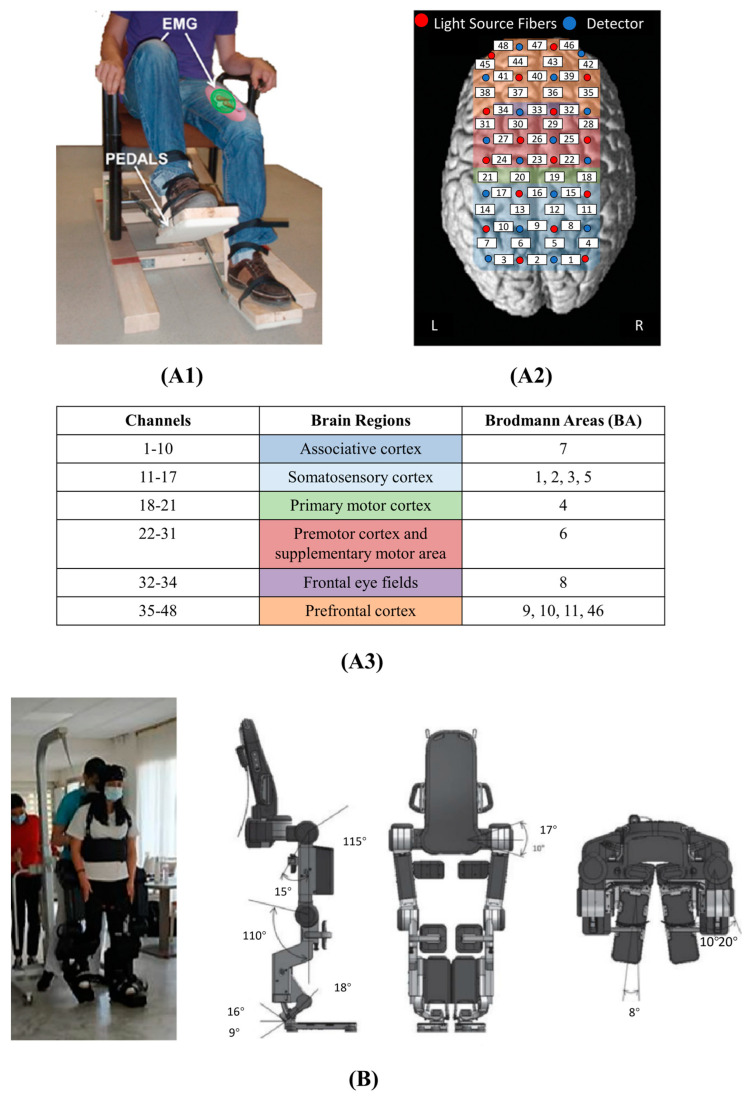
Examples of implementing fNIRS measurement in BCI systems for lower limb rehabilitation. (**A**) The pilot study of fNIRS-BCI gait rehabilitation conducted by Rea et al. Experimental setup. (**A1**) Mechanical pedals used to execute active hip movements while sitting on an armchair. EMG electrodes were positioned along muscle fibers of the femoris quadriceps and sartorius muscles of both legs. (**A2**,**A3**) Representation of optodes’ location and the corresponding anatomical location of each channel. These figures are taken with permission from [33]. (**B**) The implementation (left) and the mechanical design (right) of the Atalante^®^exoskeleton (Wandercraft company, Paris, France). This figure is taken and modified with permission from [64].

One of the pilot studies of fNIRS-BCI gait rehabilitation was conducted by Rea et al. [33]. During the preparation of hip movement, fNIRS signals (corresponding brain area in Figure 6(A2,A3)) were acquired with EMG activity recorded to ensure only tonic activity existed instead of actual movements (Figure 6(A1)). The authors noted that the conventional method of determining the coefficients of the linear relationship between HbR and HbO, based on the assumption of temporal stability, may not be appropriate for patients with neurovascular alterations, as it may result in contaminated data. A classifier based on LDA was employed and the best performance came from HbT signal changes in the posterior parietal cortex (PPC) and premotor cortex (PMC). The results highlighted the critical role of PMC and PPC which was in line with previous findings and demonstrated the feasibility of fNIRS for assessing and monitoring gait status for both healthy participants and patients with stroke. Presently, BCI development has played a vital role in investigating brain dysfunction disorders and musculoskeletal gaits [50]. Regarding robot-assisted gait training (RAGT), fused EEG-fNIRS provides a detailed insight into how locomotor control and gait recovery are characterized by brain signals. Khan et al. presented an fNIRS-based BCI framework for the control of prosthetic legs which is intended for the rehabilitation of patients suffering from locomotive disorders in 2018 [4]. The gait cycle was initiated and stopped with fNIRS signals indicating walking and resting intentions, while a non-linear proportional derivative computed torque controller (PD-CTC) with compensation for gravity was employed to control the torques of the hip and knee joints in order to minimize positional error.

A recent study conducted in 2022 explored the correlation between cortical activation and effort expended during exoskeleton-mediated gait at varying levels of physical assistance in healthy individuals (Figure 6B) [64]. The results, as indicated by the levels of HbO and HbR, with a smaller number of significant channels for HbR, showed only a minimal difference in cortical activation between the assisted conditions and the rest state. On the other hand, widespread and bilateral cortical activation was observed during the two unassisted conditions, providing support for the hypothesis that there exists a relationship between cortical activation and effort level during gait.

All these discoveries and implementations have the potential to optimize neurological rehabilitation techniques that induce neuroplasticity. The effectiveness of rehabilitation can be measured based on the level of precision with which the patients can imitate the movements of a healthy individual [36], and this frequently relies on the utilization of standardized assessments, such as the timed up and go (TUG) test [82]. In conclusion, while significant progress has been made in translational applications by various research groups, the ultimate challenge lies in transforming these complex protocols into user-friendly, cost-effective, and integrated systems that can be utilized in a home environment [4]. To achieve this goal, it is necessary to consider improving selection techniques for the most crucial brain regions to monitor, while ensuring accuracy through the utilization of minimal data, leading to faster response times. Additionally, determining the optimal configuration of brain activity, specifically the design of the training task that generates reliable control commands, remains a challenge [12]. Finally, establishing a more intuitive connection between the detected signals and machine commands can improve the ergonomics of the process and enhance the efficiency of the training.

## 6. Discussion

### 6.1. Challenges and Opportunities of Hybrid fNIRS-EEG BCIs in Motor Rehabilitation

fNIRS has demonstrated high potential as a neurorehabilitation tool for monitoring the progress of patients with regards to motor and cognitive functioning over time [63]. One limitation from fNIRS is the inherent delay in the hemodynamic response, which could result in untimely feedback for the user so as to bias the efficiency of MI or ME training. This disadvantage can potentially be remedied by integration with EEG. Additionally, while challenging, using the detection of the initial dip of the functional hemodynamic response as a supplementary feature as well as the larger and slower peak hemodynamic response may facilitate faster functional hemodynamic responses than standard fNIRS signal processing procedures [21]. The use of hybrid fNIRS-EEG BCI systems was found to be superior to the use of a single modality, due to: reducing motion artifacts and improving the reliability and robustness of signal interpretation [28]; enhancing classification accuracy of distinct conditions such as rest and task [23,24]; applying one modality as a switch for the other [42]; and utilizing the data acquired from one modality to detect motion artifacts and remove them in another, especially in MI-task-based BCIs. [24,25,40,42].

However, clearly, there is still some room for the improvement of fNIRS-EEG BCIs in motor rehabilitation. First, a fully integrated fNIRS-EEG BCI hardware with strong wearability, high tolerance of motion artifacts, stable signal quality, elegant ergonomic design, and real-time long-term sampling capability are demanded, which could enable motor rehabilitation outside of laboratory/hospital environments. Moreover, the correlation of fNIRS and EEG is not yet fully understood from a data processing prospective, and the challenges of the combined approach arise. Particularly, electrical and hemodynamic signals are not necessarily coupled: on the one hand, physiological processes (e.g., neurotransmitter synthesis) can result in hemodynamic alterations without any accompanying electrophysiological activity; on the other hand, changes in metabolic activity may not be observable if the corresponding EEG activity is short-lived and transitory [28]. The parallel processing and timing synchronization is another concern due to different intrinsic response times of fNIRS and EEG. New features selection and classification algorithms for the immediate detection of hemodynamic changes [12,44] could potentially be a solution, and additional dimensionality reductions may need to be performed so as to balance the information retrieval and computational cost.

In addition, the absence of a standardized experimental protocol has hindered direct comparisons across different signal processing and analysis methods. In the process of composing this review, the authors have noted that it is highly challenging to furnish a performance comparison of key parameters such as accuracy and response time. This limitation was not attributed to the absence of pertinent information in the studies but rather to the dissimilarities in research methodologies, such as differences in tasks, control groups, and real-time/offline processing. To address this issue, it is advised to provide a comprehensive account of all technical aspects of the experiment, including hardware and software specifications whenever possible.

### 6.2. Future Prospects—BCI + ‘X’ for Motor Rehabilitation

MI-BCI assistive devices, such as FES, could couple the activities between the brain and muscle, thereby enhancing brain activity patterns by reconstructing the functional pathway for the affected limb and even restoring the patient’s motor ability [3]. The efficacy of the desired movement relies on the design of the system and the training sessions provided to the patients on the use of the BCI-FES system.

Additionally, VR simulation environments can be employed to provide more realistic feedback for users [80]. The combination of VR and BCI offers new possibilities for enhancing user experiences, enabling more natural and intuitive interactions, and exploring novel paradigms in the field of motor rehabilitation.

Another potential avenue for rehabilitation involves incorporating feedback from cortical activation patterns, as measured by the fusion of fNIRS and EEG, to identify areas of hypo- or hyperactivity, and thus guide non-invasive brain stimulation protocols [28], such as transcranial electrical stimulation (tES), which applies a weak current through the scalp to alter brain excitability and network dynamics.

## 7. Conclusions

The integration of fNIRS and EEG remains a complex challenge, despite the technical advancements made in the areas of signal processing and data synchronization. Nevertheless, recent advancements in fNIRS/EEG devices and novel DL algorithms have shed light on the potential for using hybrid fNIRS-EEG BCIs in motor rehabilitation in a safer and more effective manner. The ultimate goal of a direct BCI is to help patients with severe motor disabilities to effectively interact with their surroundings through external devices, such as computers, text-to-speech convertors, assistive appliances, and neural prostheses, as well as helping them restore their own motor ability with rehabilitation training in almost any environment. With the recent advancements in wearable technologies, AI-enabled processing, product design, and user interface, along with the high demands and drives from rehabilitation specialists and patients, it is expected that next-generation AI-empowered, multimodal BCIs will become readily available within the next few years, which could make motor rehabilitation more effective and practical, and could then dramatically accelerate the growth of at-home rehabilitation, personalized healthcare, and beyond.

## Figures and Tables

**Table 1:** Signal processing methods in reviewed fNIRS-based BCI studies. (★ stands for clinical application).

Ref. (★ Clinical)	Feature Extraction (FE)/Channel Selection (CS)	Features	Classifier
★ [29]	-	Median of HbO, HbR	ANOVA
[30]	FE: GLM	HbO, HbR	SVM
[31]	FE: Linear-combination-based	HbO, HbR	HMM
[32]	FE: PR algorithm	HbO	SVM, ANN
★ [33]	CS: Individual-based T-map	Mean of HbT	LDA
★ [34]	-	HbO, HbD	LDA, SVM
[35]	FE: Convolutional filters	HbO, HbR	SVM, ANN, CNN
★ [36]	-	HbO, HbR (mean, peak, variance, slope, kurtosis, skewness)	k-NN, QDA, LDA, Naïve Bayes, SVM
★ [37]	CS: CNR	HbO, HbR (mean, slope)	LDA
[38]	-	HbO, HbR, HbT, HbD (mean, peak, variance)	ML: SVM, MLPNN, PBL-McRBFN DL: CNN
★ [39]	-	HbO, HbR (mean, peak, variance, kurtosis, skewness)	ML: k-NN, SVM, LDA DL: CNN, LSTM, Bi-LSTM

**Table 2:** Signal processing methods in reviewed fNIRS-EEG-based BCI studies. (★ stands for clinical application).

Ref. (★ Clinical)	Feature Extraction (FE)/Channel Selection (CS)	fNIRS Features	EEG Features	Classifier
[23]	-	HbO, HbR	µ, β-band	LDA
[24]	-	HbO, HbR	α, β-band	LDA
★ [40]	-	HbO, HbR	α, β-band (peak)	SVM
[41]	-	HbO and HbR (mean)	β-band (mean, peak)	LDA
[42]	-	HbO	δ, θ, α, β-band	SVM
[43]	FE: JMI	HbR, HbO, HbT, HbD	μ, β-band (time-phase-frequency features)	ELM
[44]	FE: CSP	HbO, HbR (mean, slope)	μ, β-band	LDA
[45]	CS: GLM FE: PCA (fNIRS), DWT(EEG)	HbO, HbR, initial dip	DWT (approximation coefficients)	SVM
[46]	-	HbO, HbR	μ, β-band	SVM, LDA, DNN
★ [47]	FE: CSP	HbO, HbR (peak, peak latency, integral area)	α, β-band	ANOVA, SVM
[48]	-	HbO, HbR	α, β-band	CNN-LSTM
[49]	-	HbO, HbR (mean)	δ, θ, α, β-band (mean)	DNN, CNN, CNN-LSTM, MCFHNet

## Data Availability

Not applicable.

## Supplementary Materials

The following supporting information can be downloaded at: https://www.mdpi.com/article/10.3390/bioengineering10121393/s1, Table S1. Signal pre-processing pipelines in reviewed fNIRS-based BCI studies; Table S2. Signal pre-processing pipelines in reviewed fNIRS-EEG-based BCI studies.

## Abbreviations

ANN	artificial neural network
ANOVA	analysis of variance
BCI	brain–computer interface
Bi-LSTM	bidirectional long short-term memory
BMI	brain–machine interface
CAR	common average reference
CNN	convolutional neural networks
CNR	contrast-to-noise ratio
COD	cerebral oxygen exchange
CSP	common spatial patterns
CWT	continuous wavelet transforms
DL	deep learning
DNN	deep neural networks
DOT	diffuse optical tomography
DWT	discrete wavelet transforms
EEG	electroencephalograph
ELM	extreme learning machines
ELU	exponential linear unit
EMA	exponential moving averages
EMG	electromyography
ERD	event-related desynchronization
ERPs	event-related potentials
FES	functional electrical stimulation
FIR	finite impulse response
fNIRS	functional near-infrared spectroscopy
FWHM	full-width-half-maximum
GLM	general linear model
HbO	oxyhemoglobin
HbR	deoxyhemoglobin
HMM	hidden Markov model
hrf	hemodynamic response filter
IA	instantaneous amplitude
ICA	independent component analysis
IF	instantaneous frequency
IP	instantaneous phase
JMI	joint mutual information
k-NN	k-nearest neighbor
LDA	linear discriminant analysis
LSTM	long short-term memory
MACD	moving average convergence/divergence
MCF	multi-channel fusion
MCFHNet	multi-channel fusion hybrid network
MDL	minimum description length
ME	motor execution
MI	motor imagery
ML	machine learning
MLP	multilayer perceptron
MLPNN	multilayer perceptron neural network
NMES	neuromuscular electrical stimulation
PBL-McRBFN	projection-based learning in a meta-cognitive radial basis function network
PCA	principal component analysis
PD-CTC	proportional derivative computed torque controller
PMC	premotor cortex
PPC	posterior parietal cortex
PR	polynomial regression
QDA	quadratic discriminant analysis
RAGT	robot-assisted gait training
RCNN	recurrent convolutional neural network
SDS	source-detector separation
SMR	sensory motor rhythm
SVM	support vector machines
tES	transcranial electrical stimulation
TSC	time-series classification
WCs	wavelet coefficients
WT	wavelet transform
