# Supplementary material for: fNIRS-EEG BCIs for Motor Rehabilitation: A Review
Source: Bioengineering (Basel). 2023 Dec 6;10(12):1393. doi: 10.3390/bioengineering10121393 (PMC10740927; doi:10.3390/bioengineering10121393)
Supplement: Supplementary file 1 [file bioengineering-10-01393-s001.zip › bioengineering-2663484-supplementary.pdf]

bioengineering

MDPI

Supplementary Material

fNIRS-EEG BCIs for Motor Rehabilitation： A Review

Jianan Chen†, Yunjia Xia†, Xinkai Zhou, Ernesto Vital, Alexander Thomas, Rui Loureiro, Robert Cooper, Tom Carlson, Hubin Zhao*

* Correspondence: Hubin Zhao: hubin.zhao@ucl.ac.uk

1. Supplementary Tables

Supplementary Table S1. Signal pre-processing pipelines in reviewed fNIRS-based BCI studies

Author/Years (★clinical)	Pre-processing
★Holper et al., 2010 [29]	• 7th order Chebyshew low-pass
Schurholz et al., 2012 [30]	• Wavelet-MDL;<br>• zero-phase Chebyshev type II low-pass filter (0.5 dB ripple, 40 [illegible]
Zimmermann et al., 2013 [31]	• 2nd order Chebychev type II low-pass filter (40 dB attenuation, 0.5 [illegible]
Thanh Hai et al., 2013 [32]	• Savitzky–Golay filter
★ Rea et al., 2014 [33]	• wavelet-MDL;<br>• Gaussian low pass filter with 4-[illegible]
★ Lee et al., 2017 [34]	• 4th order Butterworth band-pass
Trakoolwilaiwan et al., 2017 [35]	• DWT;<br>• 10-level wavelet decomposition with a Daubechies mother func-[illegible]
★ Khan et al., 2018 [36]	Implemented 6 different filters for performance comparison:<br>• Kalman Filter;<br>• Wiener Filter;<br>• Gaussian Filter;<br>• Hemodynamic response filter;<br>• Band-pass filter;<br>• [illegible]
★ Lee et al., 2019 [37]	• MACD filter
A et al., 2020 [38]	• 4th order Butterworth band-pass filter (0.01-0.2 Hz);
★ Hamid et al., 2022 [39]	• low-pass filter (0.5 Hz);<br>• high-pass filter (0.01 Hz)

Supplementary Table S2. Signal pre-processing pipelines in reviewed fNIRS-EEG-based BCI studies

Author/Years (★clinical)	fNIRS Pre-processing	EEG Pre-processing
Leamy et al., 2011 [23]	• low-pass filter (0.5 Hz);<br>• high-pass filter (0.01 Hz)	• high-pass filter (1 Hz)
Fazli et al., 2012 [24]	• 3rd order Butterworth low-pass filter	• band-pass filter;<br>• CSP-based filter
★ Khan et al., 2014 [40]	• Gaussian low-pass filter;<br>• wavelet transform	• band-pass filter (4-35 Hz)
Jawad Khan et al., 2014 [41]	• Gaussian low-pass filter;<br>• wavelet transform	• band-pass filter
Koo et al., 2015 [42]	• EMA filter	• Butterworth filter;<br>• CSP-based filter
Yin et al., 2015 [43]	• 5th order Chebyshev type II low-pass filter (0.1 Hz)	• 5th order Chebyshve type II low-pass filter (125 Hz);<br>• band-pass filter (5 to 45 Hz);<br>• Laplacian filter
Buccino et al., 2016 [44]	• 4th order IIR Butterworth filter (0.01-0.2 Hz);<br>• High-pass filter	• 4th order IIR Butterworth band-pass filter
Li et al., 2017 [45]	• 4th order Butterworth band-pass filter (0.01-0.2 Hz)	• 3rd order Butterworth band-pass filter (1-45 Hz)
Chiarelli et al., 2018 [46]	-	• zero-phase 2nd order Digital Butterworth filter (8-30Hz)
★ Wang et al., 2019 [47]	• band-pass filter (0.01-0.2 Hz);<br>• moving average outlier removal	• band-pass filter (0.5-100 Hz);<br>• band-stop filter (50 Hz);<br>• CAR
Ghonchi et al., 2020 [48]	• band-pass filter (0.01-0.5Hz)	• band-pass filter (0.5-35Hz)
Chen et al., 2022 [49]	• band-pass filter (0.01-0.2Hz)	• 3rd-order Butterworth filter (0.5-35Hz)

Disclaimer/Publisher's Note: The statements, opinions and data contained in all publications are solely those of the individual author(s) and contributor(s) and not of MDPI and/or the editor(s). MDPI and/or the editor(s) disclaim responsibility for any injury to people or property resulting from any ideas, methods, instructions or products referred to in the content.
